# Facilitation between woody and herbaceous plants that associate with arbuscular mycorrhizal fungi in temperate European forests

**DOI:** 10.1002/ece3.2757

**Published:** 2017-01-24

**Authors:** Stavros D. Veresoglou, Monika Wulf, Matthias C. Rillig

**Affiliations:** ^1^ Institut für BiologieFreie Universität BerlinBerlinGermany; ^2^ Berlin‐Brandenburg Institute of Advanced Biodiversity ResearchBerlinGermany; ^3^ Institute of Land Use SystemsLeibniz Centre for Agricultural Landscape Research (ZALF)MünchebergGermany

**Keywords:** Arbuscular mycorrhiza, Glomeromycota, herbaceous plant communities, mycorrhizal mediation, plant–soil (below‐ground) interactions, temperate European forests, vegetation plots

## Abstract

In late‐successional environments, low in available nutrient such as the forest understory, herbaceous plant individuals depend strongly on their mycorrhizal associates for survival. We tested whether in temperate European forests arbuscular mycorrhizal (AM) woody plants might facilitate the establishment of AM herbaceous plants in agreement with the mycorrhizal mediation hypothesis. We used a dataset spanning over 400 vegetation plots in the Weser‐Elbe region (northwest Germany). Mycorrhizal status information was obtained from published resources, and Ellenberg indicator values were used to infer environmental data. We carried out tests for both relative richness and relative abundance of herbaceous plants. We found that the subset of herbaceous individuals that associated with AM profited when there was a high cover of AM woody plants. These relationships were retained when we accounted for environmental filtering effects using path analysis. Our findings build on the existing literature highlighting the prominent role of mycorrhiza as a coexistence mechanism in plant communities. From a nature conservation point of view, it may be possible to promote functional diversity in the forest understory through introducing AM woody trees in stands when absent.

## 1 Introduction

Plant communities often contain individuals belonging to different life forms. Sutherland et al. (2013) identified as one of 100 fundamental questions in ecology investigating how coexistence of plants with different life forms is possible. Temperate forests are a well‐known example of a habitat where plants differing in their life forms coexist; several hypotheses have been proposed to explain the coexistence of woody and herbaceous plants in such forests (Nakashizuka, 2001). An overlooked feature that could influence this coexistence relates to the mycorrhizal associations they form. Mycorrhizas are trophic symbioses (with a variety of other nontrophic functions) formed between fungi and plant roots (Smith & Read, 2008). As they have pervasive effects on plant host fitness, the presence of mycorrhizal fungi can facilitate the successful establishment and proliferation of their plant hosts (Bergelson & Crawley, 1988). The relevance of mycorrhiza for plant species diversity has been thoroughly demonstrated (Klironomos et al., 2011). However, the extent to which mycorrhizas influence plant diversity seems to depend on the degree of mycorrhizal dependency (Grime, Mackey, Hillier, & Read, 1987; Bergelson & Crawley, 1988; O'Connor, Smith, & Smith, 2002). There are several obvious ways through which mycorrhiza can mediate plant coexistence. For example through facilitating the establishment of seedlings, amplifying allelopathic interactions and interspecific negative feedbacks (many of which are reviewed in Hart, Reader, & Klironomos, 2003; but also in more recent literature, e.g., Veiga, Howard, & van der Heijden, 2012; Badikova et al., 2013). Here, we refer to the possibility that donor plants, by providing mycorrhizal access or inoculum to other plants, promote establishment and survival of these target plants, as the “mycorrhizal mediation hypothesis.” To the best of our knowledge, the possibility that mycorrhizas mediate coexistence of plants differing in life forms by this general mechanism has never been tested.

There are multiple types of mycorrhiza, the two most widespread of which are arbuscular mycorrhizas (AM), prevalent in herbaceous plants, and ectomycorrhizas (ECM), prevalent for example in temperate woody plant species (Smith & Read, 2008). Each of these two mycorrhizal types involves a distinct set of fungi (Smith & Read, 2008). The vast majority of woody plants and consequently biomass in European temperate forests consists of ECM plants (Smith & Read, 2008). The respective (quartile) figures for herbaceous plants were 3%–7% (ECM) and 40%–49% (AM). The mycorrhizal mediation hypothesis has been tested several times with a focus on ECM plants and in most cases the data supported the hypothesis (e.g., Dickie, Guza, Krazewski, & Reich, 2004; Richard, Selosse, & Gardes, 2009; Teste et al., 2009; Moeller, Dickie, Peltzer, & Fukami, 2015) which appears to be due to ECM propagule limitation (Dickie et al., 2004; Dickie, Davis, & Carswell, 2012) . Despite our awareness that AM propagules decline in the forest compared to herbaceous systems (Fisher & Fulé, 2004), we know much less about the possibility that mycorrhizal mediation exists in temperate forests with regard to AM fungi. Becklin, Pallo, and Galen (2012) experimented with alpine perennial plants to show that the root colonization rate of herbaceous plants declined in the presence of willow plants when these had been colonized by ECM fungi. Because the dominant trees in temperate forests are colonized by ECM fungi and AM propagules are rare, we believe that the mycorrhizal mediation with regard to AM‐associating herbaceous plants should be of higher ecological significance than with regard to ECM‐associating herbaceous plants. With the possible exceptions of van der Heijden (2004), van der Heijden and Horton (2009) and Varga and Kytöviita (2016), to the best of our understanding, the mycorrhizal mediation hypothesis has never been tested so far with a focus on AM associations.

Despite the high number of studies describing forest ecosystems, there are still several open questions in forest ecology (Sutherland et al., 2013). Even though the herbaceous plants account for a small fraction (often <1%) of stand biomass, they represent a considerably higher fraction of forest net primary productivity and litter production (Gilliam, 2007). Herbaceous plants in forests thus are important regulators of nutrient cycling and overall ecosystem functioning (Welch, Belmont, & Randolph, 2007). Moreover, the concept that herbaceous communities in the understory of temperate forests are only influenced by the woody plants but do not influence them in turn has been challenged in the literature. There is evidence that some herbaceous plants may be exceptionally efficient competitors for nutrients and can influence the fitness of trees (Lyon & Sharpe, 2003). Most importantly, herbaceous plants may influence the successional trajectory of a stand or during regeneration through either suppressing (Horsley, 1993; George & Bazzaz, 2003; Gilliam, 2007) or facilitating (van der Heijden, 2004) seedlings of woody species. Finally, herbaceous plants comprise approximately 80% of plant species diversity in forests and become extinct at considerably higher rates than woody plants (Gilliam, 2007). Given our increasing awareness that plant communities are rarely if ever functionally redundant (Isbell et al., 2011), it is important to understand herbaceous understory plant communities for effective conservation.

Even though the main focus in managing European temperate forests is on maximizing timber production (Jonsson, Pe'er, & Svoboda, 2015), achieving a high biodiversity can promote a wide range of ecosystem services (Mace, Norris, & Fitter, 2011) and is desirable (Brockerhoff, Jactel, Parrotta, Quine, & Sayer, 2008). A possible way to improve forest management practices consequently aligns with achieving a better understanding of biotic interactions in the forest (Araújo & Luoto, 2007). There is a considerable number of studies describing temperate forests which shows that biotic interactions represent key modulators of mature tree mortality (Das, Battles, Stephenson, & van Mantgem, 2011), decomposition rates and nutrient cycling (Rouifed, Handa, David, & Hättenschwiler, 2010), and seedling recruitment (Montgomery, Reich, & Palik, 2010; Muhamed, Touzard, Le Bagousse‐Pinguet, & Michalet, 2013). These facts highlight the need to focus more on the role of biotic interactions in the understory (Nilsson & Wardle, 2005). Gilbert and Lechowicz (2004) examined the relative importance of niche‐based versus neutral‐based processes in shaping the understory in a forest in Canada, finding that neutral theory was of limited explanatory power in their system. In that study community structure of the understory was considered in isolation from that of the tree canopy (Gilbert & Lechowicz, 2004). Because herbaceous plants can benefit from the canopy in a number of ways including hydraulic lifting (Ishikawa & Bledsoe, 2000) and symbiotic interplant transfer of nutrients (van der Heijden, 2004; Simard, 2009), the effects of canopy on understory could be more pertinent than found in Gilbert and Lechowicz (2004). We further know that under specific conditions canopy trees may exert strong negative effects on the recruitment of juveniles belonging to the same species (Johnson, Beaulieu, Bever, & Clay, 2012). Understanding the extent to which the community structure of herbaceous plants is dependent on that of the trees may be useful to develop conservation tools and facilitate environmental monitoring (e.g., Grandin, 2004). A prevalent pathway of indirect canopy–understory interactions is the symbiotic pathway (van der Heijden, 2004; Simard, 2009). Because most woody plants in temperate forests associate with ECM fungi, availability of ECM propagules should rarely be a problem and the only case where mycorrhizal mediation might be likely is with regard to AM‐associating plants.

Here, we tested whether and to what extent woody plants that associate with AM fungi facilitate the establishment of herbaceous plants of compatible mycorrhizal types in agreement with the mycorrhizal mediation hypothesis. Different mycorrhizal types may occupy different ecological niches, for example be distributed at different soil pH ranges. AM‐richness declines considerably in habitats with low soil pH (Kohout et al., 2015), whereas ECM trees (and their symbionts) often occur in low pH environments (Smith & Read, 2008). In particular, the mycorrhizal‐associated nutrient‐economy model (Phillips, Brzostek, & Midgley, 2013) predicts that the environment represents a decisive factor for the type of mycorrhiza that establishes. It is likely that effects attributed to mycorrhizal mediation are actually driven by differences in physiological tolerance in the two types of mycorrhiza. For this reason, we tried to correct in our analysis for possibly confounding abiotic and biotic factors. We narrowed our focus on AM plants because of the dominant role ECM plants play in temperate forests which render them unlikely to be limited by ECM fungal propagules (Peay, Bruns, Kennedy, Bergemann, & Garbelotto, 2007). We hypothesized that AM herbaceous plants would be less abundant and would be represented by fewer species in forest stands where there are few or no woody plant species that associate with AM fungi (mycorrhizal mediation hypothesis).

## 2 Materials and Methods

### 2.1 Sources of data

**Figure 1:**
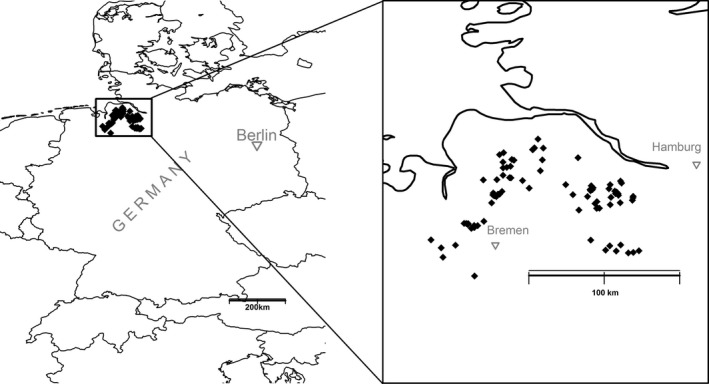
Map of Germany with the locations of the forest plots considered in our analysis highlighted with black rhombs

We used a dataset originating from Wulf (1992). The dataset consists of the plant community structure at 415 plots, ranging in size from 100–400 m^2^, located in 77 mixed broadleaf forests in the Weser‐Elbe region in northwestern Germany (Figure 1). All forest plots were located in ancient forests (habitat continuity for over 200 years—Naaf & Wulf, 2010). The stands had already not been intensively managed at the time of the survey (1992) for several decades. Plant community data were encoded into the extended Braun‐Blanquet scale (Braun‐Blanquet, 1964), and plot information was supported by coordinates and some basic characterization of the soil properties of the plots. Plants in the dataset were preclassified as either woody or herbaceous. Braun‐Blanquet values were converted to abundance values with the coefficients proposed by van de Maarel (2007). Ellenberg values for the herbaceous plant species in Germany were retrieved for soil reaction (called Ellenberg R) and usually highly correlated with the soil pH, an indicator of nitrogen but also overall fertility, called Ellenberg N, and moisture, called Ellenberg F (Ellenberg et al., 2001). The Ellenberg indicator values represent a system which uses plants as bioindicators of environmental conditions. It consists of a table where for each plant species and environmental parameter a value from 1 to 9 is assigned, and a value describing how narrow the tolerance estimates for the species are (Ellenberg et al., 2001). We used these values to infer environmental values through a maximum entropy approach (Guiasu & Shenitzer, 1985). In brief, we worked separately with each indicator variable, and for each plot we calculated an average of the indicator values for the species found at the site weighted by the abundance of each plant species. To produce a more accurate estimate, we excluded plants with an indifferent occurrence pattern (i.e., poor indicator species that are found over a wide range of the environmental variable considered and whose occurrences are less informative than for other species) or for which estimates were missing. The resulting weighted indicator value was treated as a metric of the respective environmental variable. To assess the reliability of the technique for the subset of plots where pH had been assayed (126 plots), we compared the pH values that were measured in Wulf (1992) with those that we estimated through the weighted‐mean Ellenberg value approach. Information on the mycorrhizal status of plants was obtained from Wang and Qiu (2006). As an alternative source of mycorrhizal status, we used the data in Hempel et al. (2013).

### 2.2 Statistical analysis

We scored mycorrhizal status of the plants by giving a “zero” to plants that were reported as not associating with AM and a “1” to those for which the only reported mycorrhizal status was AM. For plants that were reported as nonmycorrhizal or as forming a different type of mycorrhiza in addition to AM, we assigned a weight of “0.5,” but we also tried different weights (sensitivity analysis). Consideration of plants with multiple mycorrhizal states was particularly important for woody plants as there were only few putatively only‐AM woody plants. In the rare cases (35 herbaceous but none of the woody plant species out of a total of 220 plant species) where the mycorrhizal status was not known, we excluded these species from our analysis.

The predictor variable in our analyses was relative abundance of AM woody plants. Possible herbaceous community responses to relative abundance of AM woody plants can include changes in plant species richness or in overall abundance of AM herbaceous plants, or both. In our statistical analysis, we attempted to independently address each kind of response. For both analyses, herbaceous plant community data were converted to a single response variable (i.e., relative richness or relative abundance of AM herbaceous plants) and were subsequently analyzed with univariate statistics. To account for spatial autocorrelation constraints in our data, we introduced autocorrelation structures in our models so that the criterion of optimum model parsimony was achieved. To assess model parsimony we used the Akaike information criterion. The autocorrelation structure that performed best in these models was an exponential autocorrelation structure. Whenever the assumptions of homoscedasticity and normality of the residuals were not met, we assessed relationships with a nonparametric test—the Kendall *Tau* test. Because mycorrhizal mediation effects of AM woody plants were likely to be limited to environments dominated by ECM and nonmycorrhizal plants, we explored our relationships with segmented regression.

For our analysis on relative species richness of AM understory plants (i.e., the proportion of species in the understory that were arbuscular mycorrhizal), our preliminary tests revealed that heteroscedasticity was a potential issue. For this specific analysis, we therefore used the nonparametric Kendall *Tau* test to infer the presence or absence of a relationship with the observed relative abundance of AM woody species. Here, we deemed correcting for spatial autocorrelation unnecessary due to the high number of entries and because of the nature of the analysis that relied on rankings (nonparametric analysis). Because we could identify an overall positive relationship between the woody plant community on the herbaceous stratum throughout the arbuscular mycorrhizal availability gradient, we did in this case not attempt to fit a segmented regression model.

With regard to our analysis of relative abundance of the AM herbaceous plants, we ran a segmented regression to identify a breakpoint beyond which further increases in the relative abundance of AM woody plants had little effect on the herbaceous community. Based on the rationale we present above, we focused our analyses on the forest plots for which woody‐AM relative abundance was before that breakpoint. In these analyses, we considered models that accounted for spatial autocorrelation and models that did not, which gave comparable results. To account for spatial dependencies, we used an exponential correlation structure with a nugget effect as implemented through the corExp directive in the package nlme (Pinheiro, Bates, DebRoy, & Sarkar, 2013).

To account for confounding effects of environmental variables, we carried out a path analysis. Preliminary testing revealed that any path models that considered more than three variables had a poor fit and violated the null assumption of the Chi‐square test. We thus tested five structural equation models (SEM) consisting of relative abundance data for woody and herbaceous plants and either of the three abiotic variables (SEM1—Ellenberg R values, SEM2—Ellenberg N values, SEM3—Ellenberg F values) or the biotic factor (log response ratio of herbaceous vs. woody plant abundance) and a single model where we investigated the combined effects of the most parsimonious abiotic predictor with our biotic one. In our SEM analyses, we did not consider spatial dependencies. Our SEMs were formulated as partial correlation analyses. A complementary conservative way to account for environmental variables was to fit a linear model with relative abundance data for the understory as dependent variable and all environmental variables (either all abiotic or all abiotic and biotic together) as predictors and then assess the fit of the relative abundance data for woody plants on the residuals of the earlier model. That way, the specific model assessed the marginal relationship (i.e., variability explained after we have fitted other parameters) between herbaceous and woody plants. All analyses were carried out in R version 3.0.2 (R Development Core Team 2012).

### 2.3 Sensitivity analyses

We carried out two different sensitivity analyses. We first tested the effect of allocating mycorrhizal weights different from 0.5 to the plants that had been classified as AM but had been also observed in a different mycorrhizal state. For this analysis, we tried all possible weighting values from zero to one with a step of 0.05. For each of these weighting values, we recalculated relative abundances in both the strata of woody and herbaceous plants and assessed the significance of the relationship between the two strata for the subset of plots for which the relative abundance of AM woody plants was lower than the identified threshold. We tested these relationships with and without accounting for spatial autocorrelation. We also used as alternative mycorrhizal status definitions those published in Hempel et al. (2013). For this analysis, we assumed the usual weighting value of 0.5 for species that have been reported capable of associating with ectomycorrhizal fungi or remaining nonmycorrhizal in addition to being found in association with Glomeromycota.

## 3 Results

Irrespective of the plant AM mycorrhizal state definitions used, from Wang and Qiu (2006), or those from Hempel et al. (2013), the relative abundance (11.31% vs. 6.34%—understory vs. canopy, *t* = 28.1, *p *<* *.001) and richness (21.9% vs. 18.7%—understory vs. canopy, *t* = 5.18, *p *<* *.001) of AM plants in the understory was higher than in the canopy. Through our maximum entropy approach, we inferred indicator values for the described plots ranging between (min, max, median) 4.2, 6.7, 5.9 for nitrogen; 4.2, 7.1, 6.3 for pH; and 5.0, 7.2, 6.0 for moisture. Soil pH estimates originating from Ellenberg values and the weighted abundance of plants in plots were comparable to those originating from in situ measurements (Fig. S1). The log response ratio of inferred abundances of herbaceous versus woody plants correlated well with the relative abundances of herbaceous AM plants (*R* = .56, *p *<* *.001).

**Figure 2:**
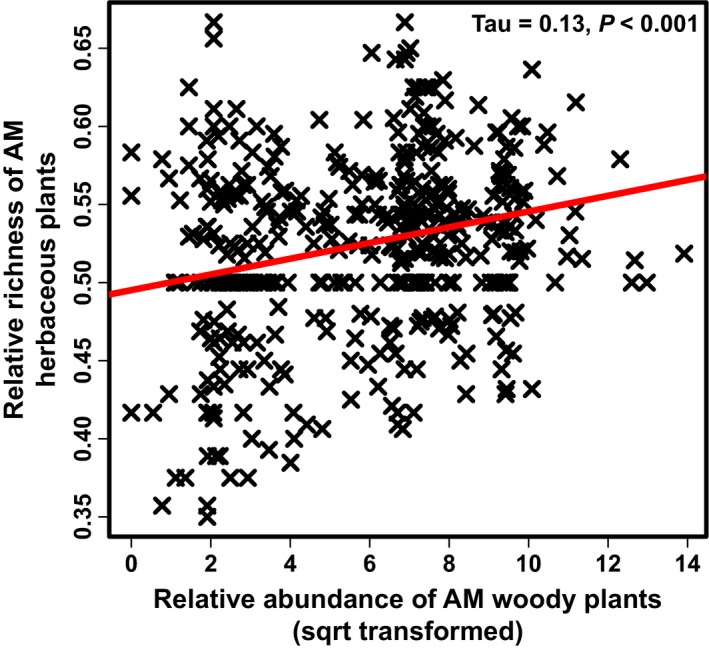
Relationship between relative abundance of AM woody plants and relative species richness of AM herbaceous plants in the understory of forests in the Weser‐ Elbe region in Germany. The red line was derived through median quantile regression for visualization purposes. Statistics presented on the top right corner of the panel are based on a Kendall correlation test. Likely spatial dependencies were not considered in the test. Note that back‐transformed relative abundances may exceed 100% as they are based on converted estimates from an extended Braun‐Blanquet scale

**Figure 3:**
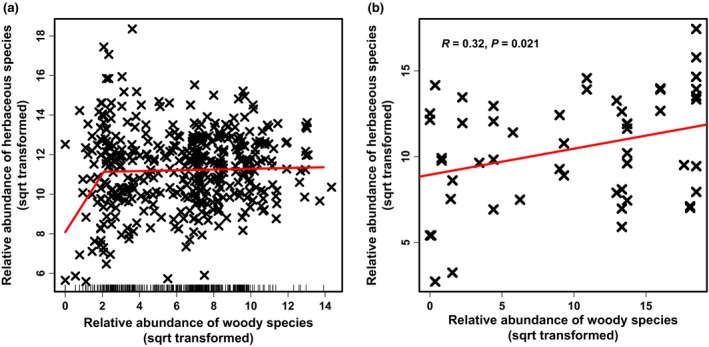
(a) segmented regression and (b) analysis of forest plots on the left side of the identified breakpoint between relative abundance of AM woody species and relative abundance of AM herbaceous plants. The analysis in panel (b) takes into consideration likely spatial dependencies and is based on a parametric linear model. (Figure will be redrawn to combine plots)

**Figure 4:**
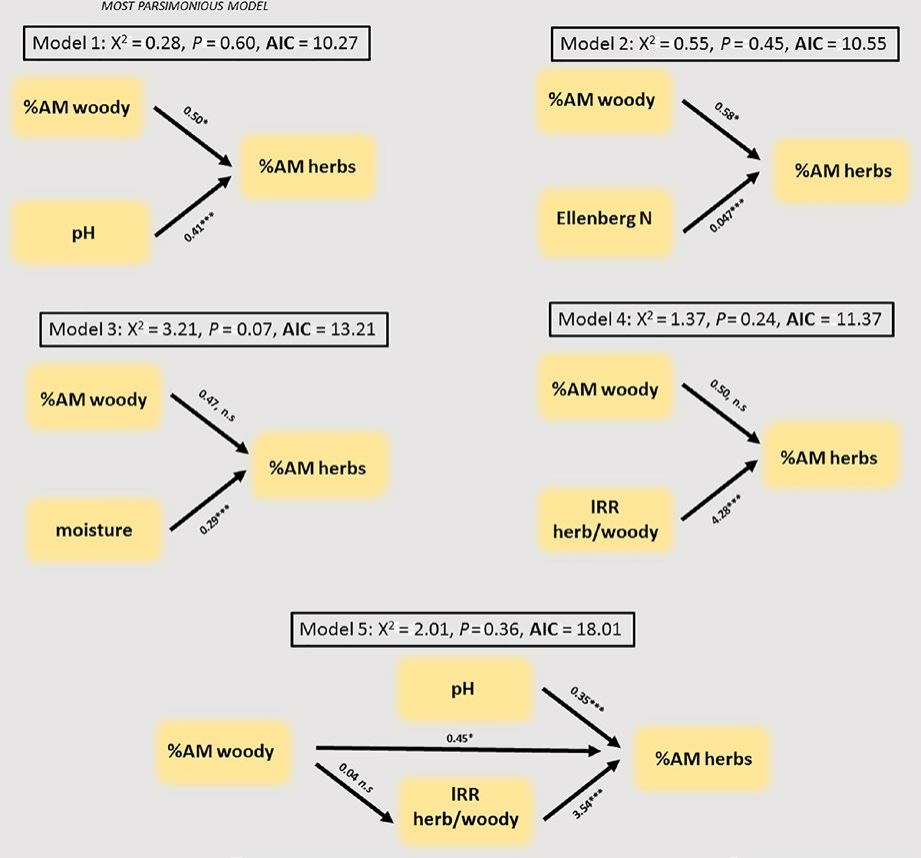
Five simple path models on relative abundance data on herbaceous and woody plants in locations with a low (as identified via a segmented regression) relative abundance of woody plants. The first four models only consider a single environmental constrain. More complex models had a worse fit (Model 5 was the most parsimonious of those we tried). The most parsimonious model was the one that considers Ellenberg pH weighted values. Values on the arrows highlight parameter estimates and their significance level (*p <* *.05, ***p <* *.001)

We found a significant positive relationship between relative species richness of AM herbaceous plants and relative abundance of AM woody plants (Figure 2). The relationship was even stronger when we used absolute richness of herbaceous species that associated with AM as a response variable (Fig. S2). Following segmented regression (Figure 3a), the relationship between the relative abundance of AM herbaceous plants and AM woody plants in the plots that had lower relative abundance of AM woody plants than the breakpoint (4.3% relative abundance) was also significant (Figure 3b). Of the five SEM models considered, the most parsimonious (smaller AIC value) was that with Ellenberg pH values as a second predictor (Figure 4). In the specific model, both the effects of Ellenberg pH and % relative abundance of AM woody plants were significant. Our linear models that evaluated whether a relationship existed between the marginal variation of herbaceous relative abundance of AM plants after correcting for abiotic environmental variables (*t *=* *2.205, *p *=* *.032) or both abiotic and biotic variables (tau = 0.19, *p *=* *.045) and the relative abundance of AM woody plants were significant (Fig. S3).

### 3.1 Sensitivity analyses

The relationship between relative abundance of woody and herbaceous AM plants was not particularly sensitive to the choice of the weight for intermediate weights (Table S1) but faded for extreme weight values. Repeating the analysis with the alternative definitions of mycorrhizal status gave comparable results (Fig. S4; Fig. S5).

## 4 Discussion

We tested the hypothesis that the relative richness and abundance of AM herbaceous plants would be lower in forest stands dominated by non‐AM trees and found evidence for this. We take these effects as evidence that AM mycorrhizal mediation is robust to the metric of plant community structure chosen. In our analyses and subsequent inference, we are treating the stratum of woody plants as an independent variable and that of herbaceous plants as a response variable. We justify this on the basis that while forest management practices in Europe control for community structure of woody plants, forests are rarely managed directly for herbaceous plants. Additionally, population demographics in the overstory exceed considerably those in the understory. These facts allow us to assume causality of the realized patterns. The positive relationship between relative abundance of AM herbaceous and AM woody plants were robust to the consideration of environmental parameters (tested by path analyses). We considered three environmental parameters, namely pH, moisture and nitrogen and a biotic variable—the inferred relative abundance of herbaceous over woody plants. Despite the large size of the dataset, our models were more parsimonious when single parameters were considered. We obtained the most parsimonious model when we fitted Ellenberg N values that reflected site fertility. Both ECM and AM plants are known to be sensitive to high nutrient availability, but this effect appears to be more pronounced for ECM plants (Hoeksema et al., 2010). The positive coefficient (describing a positive relationship) with regard to Ellenberg N indicator values that we found in our most parsimonious path model could arise from the high sensitivity of ECM associations to high site fertility conditions. Alternatively, site fertility effects could be unrelated to the functioning of mycorrhiza and be indirect as AM and ECM plants appear to have distinct site preferences. For example, Averill, Turner, and Finzi (2014) found that ECM plants are more commonly observed than AM plants in soils that contain more carbon. We are also aware that the establishment of ECM plants in late‐successional dune systems is accompanied with a steep decline in pH (Read, 1989). Other examples relate to the faster decomposition of AM‐tree‐derived litter (Cornelissen, Aerts, Cerabolini, Werger, & van der Heijden, 2001) and N and P cycling rates (Phillips et al., 2013). Despite controlling for the abovementioned factors, ours was still an observational study for which causality cannot be directly shown.

How could we explain the relationship between the relative richness and abundance of herbaceous AM plants and the respective relative abundance of AM woody plants? It is unlikely to have been due to AM‐mediated facilitation from mother plants as this has been shown so far only for conspecifics (van der Heijden, 2004). We also do not think that this pattern is caused by production of enzymes that facilitate AM establishment by AM woody plants, as there is no evidence that any AM‐specific enzymes exist. We suggest that in part the relationship we observe is due to AM‐colonized trees speeding up nutrient cycling (Phillips et al., 2013) which enables establishment of herbaceous plants that associate with AM. This (biotic) effect may be present even after accounting for observed differences in abiotic parameters and could explain the relationship between relative richness of AM herbaceous plants and that of relative abundance of AM woody species. With regard to relative abundance of AM herbaceous plants, however, there was a peak for a relatively low relative abundance threshold of AM woody plant species (4.3%) and then the relative abundance of AM herbaceous plants remained constant. We assume that this was due to AM propagule limitation (Fisher & Fulé, 2004) when the stands consist of only few AM‐associating trees which could have prevented the establishment of any herbaceous plants (and thus of a thriving herbaceous layer—i.e., strong relationship between relative abundance of AM herbaceous plants and log response ratio of abundances of herbaceous vs. woody plants). Our results supported the assumption that the relative abundance of AM woody plants was relatively low, even when AM propagules where sufficient and an AM‐fungal networked had been established. In that case, only AM herbaceous taxa that could establish that were specifically adapted to low nutrient cycling rates. This explanation could justify why our results differ with regard to relative richness and abundance for AM herbaceous plants.

Could the declined abundance of AM herbaceous plant species in stands dominated by non‐AM trees have implications for ecosystem functioning? In mycorrhizal ecology it is thought that ECM plants receive considerable more pronounced benefits from their fungal partners than AM plants do (Veresoglou & Rillig, 2014). While this is an idea that can be conceptualized only at an individual and not ecosystem scale it reflects how pronounced the functional differences between AM and ECM associations may be. ECM woody plants have been shown to have thinner roots and greater branching intensity than AM woody plants (Comas, Callahan, & Midford, 2014). Mixed AM and ECM woody forest communities could thus have a higher functional diversity and complementarity with regard to root architectural/phenological characteristics (traits) which could lead to higher ecosystem process stability (Diaz & Cabido, 2001). Moreover, ECM and AM plants have been shown to respond in radically different ways to carbon availability. For example, Brzostek, Dragoni, Brown, and Phillips (2015) experimentally removed soil carbon in plots to find lower soil organic matter degrading enzyme activity in ECM‐dominated plots whereas in AM‐dominated plots little change in enzyme activity occurred, but an overall increase in decomposition. Coexistence of ECM and AM plants could thus have a mitigating effect on stand resilience to environmental stresses. Comparable arguments for ecosystem resilience can be made from a mycological perspective due to the resulting higher microbial diversity (Bardgett & van der Putten, 2014) when both ECM and AM fungi coexist in a habitat.

The community structure of European forests has been studied extensively, particularly with regard to the phytosociological nature of the communities (Spribille & Chytrý, 2002). While the contribution of these studies to our understanding of forests is undeniable, we still lack predictive ecological tools to understand the implications of human intervention. An important finding of our study was that the relationship for the relative abundance of understory AM plants remained robust following consideration of environmental parameters. In our study, the size of our plots ranged between 100 m^2^ and 400 m^2^ which is a relatively coarse study grain. Even though we effectively controlled for abiotic dependencies in many respects the specific plot grain may be considered relatively crude to be informative for the study of biotic interactions and in our case facilitation. While this may be true for most biotic interactions, mycorrhizal facilitation arising from mycorrhizal propagule availability represents a special case of a biotic interaction which is meaningful to consider only at relatively coarse plot grains. In fact, the coarse grain of the study was an advantage because it masked to a large extent other biotic interactions; and we appropriately addressed the confounding effects of environmental filtering through correcting for environmental variables. It is becoming apparent that managing forests for maximum net primary productivity requires a high diversity of plants (Gamfeldt et al., 2013). ECM‐dominated forests are occasionally species poor to the extent that re‐establishing non‐ECM plants can be challenging (Weber, Günter, Aguirre, Stimm, & Mosandl, 2008). A possible cause could be the lack of AM propagules (e.g., Fisher & Fulé, 2004) that could support growth of a range of herbaceous understory plants (e.g., van der Heijden, 2004). Contrary to ECM systems (e.g., Kranabetter, de Montigny, & Ross, 2014), there are no studies on the threshold at which relative abundance of AM plants severely limits AM fungal propagule availability, and in our study it appears to be quite low (4.3%).

In conclusion, we here present evidence in support of the applicability of the mycorrhizal mediation hypothesis to temperate forest systems. A group of plants underrepresented in temperate forest, namely AM plants, appear to be affected by the relative abundance of AM‐forming woody species in a stand. This likely indicates that these woody plants act as AM fungal propagule source islands in these ecosystems. Our results are important from a management perspective but can also contribute to a discussion on the conditions that permit the establishment of AM plant taxa in temperate forests.

## Conflict of Interest

None declared.

## Data Accessibility

The dataset used for the study is available at http://www.givd.info/db_details.html?choosen_db=114&choose=Load.
