# Supplementary material for: Facilitation between woody and herbaceous plants that associate with arbuscular mycorrhizal fungi in temperate European forests
Source: Ecol Evol. 2017 Jan 24;7(4):1181–9. doi: 10.1002/ece3.2757 (PMC5306016; doi:10.1002/ece3.2757)
Supplement: Supplementary file 1 [file ECE3-7-1181-s001.doc]

**Supplementary Material**

Contents:

Extended materials and methods

One supplementary table

Five supplementary figures

Results from the fitted structural equation models

Extended Materials and Methods

The spatial autocorrelation function we used in our corrected for spatial dependencies model was a corExp autocorrelation structure described by the latitude and longitude of the site and a nugget effect (intercept) present.

To convert our Braun-Blanquet data to abundance data we used the following abundance values (van der Maarel 2007).

Classes Abundance

5a/5b 160

4a/4b 80

3a/3b 40

2b 20

2a 10

2m 5

1b/1a/1p 2.5

x/+b/+a/+p 1.2

+r 0.6

| Table S1. Sensitivity analysis of the effect that the weighting scheme of plants with an additional mycorrhizal status (other than AM) had on the significance of the relative abundance relationship between woody and understory plants (sites with lower woody AM relative abundance than the breakpoint) after accounting for spatial dependencies. Both models that take into consideration the spatial structure of the plots (spatial) and overlook it (naïve) were considered. | | | | | | | | | | | | | | | | | | | | | |
| --- | --- | --- | --- | --- | --- | --- | --- | --- | --- | --- | --- | --- | --- | --- | --- | --- | --- | --- | --- | --- | --- |
| weight | 0 | 0.05 | 0.1 | 0.15 | 0.2 | 0.25 | 0.3 | 0.35 | 0.4 | 0.45 | 0.5 | 0.55 | 0.6 | 0.65 | 0.7 | 0.75 | 0.8 | 0.85 | 0.9 | 0.95 | 1 |
| *P* (spatial) | 0.774 | 0.155 | **0.004** | 0.78 | 0.911 | 0.561 | 0.169 | 0.491 | **0.016** | **0.046** | **0.027** | **0.049** | **0.028** | **0.025** | 0.324 | **0.075** | 0.36 | 0.105 | 0.152 | 0.255 | **0.077** |
| *P* (naive) | 0.508 | **0.044** | **0.049** | **0.048** | 0.11 | 0.149 | 0.169 | 0.153 | **0.089** | 0.141 | **0.081** | 0.121 | 0.177 | 0.101 | 0.362 | 0.232 | 0.367 | 0.262 | 0.257 | 0.48 | 0.16 |
|  |  |  |  |  |  |  |  |  |  |  |  |  |  |  |  |  |  |  |  |  |  |


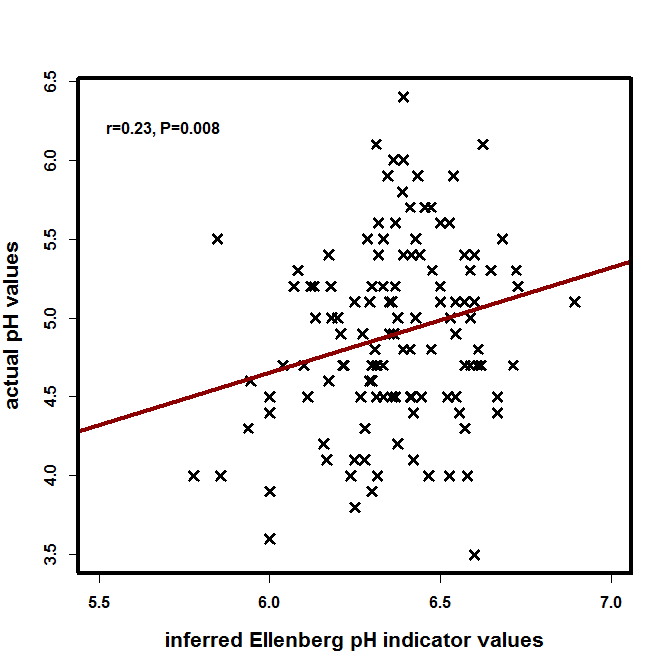


Fig S1 – Relationship between inferred and observed (actual) pH values for the sites for which information was available. Statistics are overlaid on the graph.


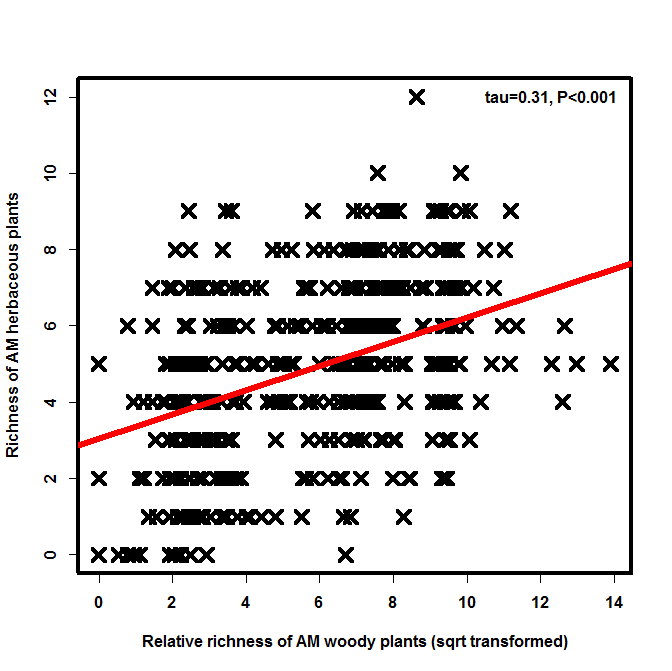


Fig. S2. Relationship between relative richness of AM woody plants and the absolute richness of AM-associating herbaceous plants. The relationship is significant with a stronger effect size than the respective relationship with relative richness of AM herbaceous plants. We believe that even though using of relative values in our correlations was more conservative, it better controleld for the variability in herbaceous cover that we observed across plots in the dataset.


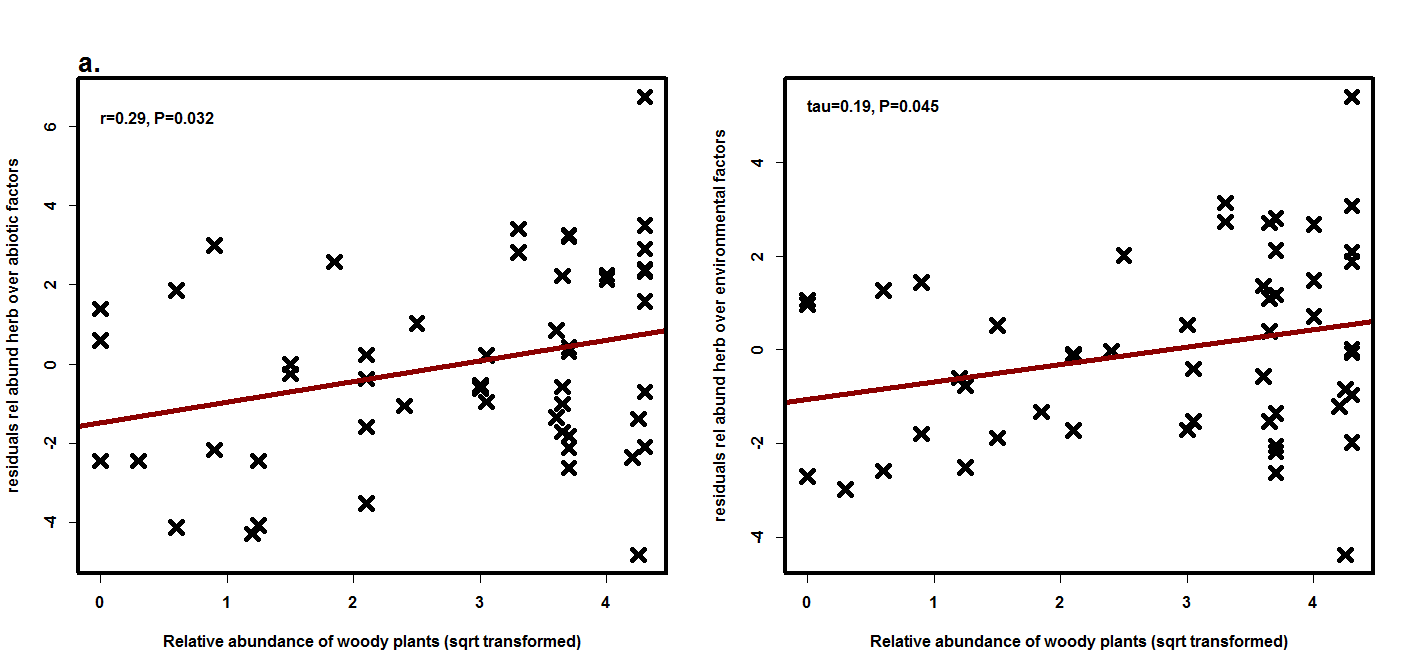


Fig. S3 Marginal relationships between relative AM woody abundance and relative AM herbaceous abundance for the subset of plots where the relative AM woody abundance was lower than the threshold of 4.3%. In (a) the model that was used to calculate the residuals accounted for three main abiotic parameters (pH, nutrients and moisture) whereas in (b) it additionally accounted for the log response ratio of herbaceous vs woody abundance in the plots. All assumptions for the analysis were met.


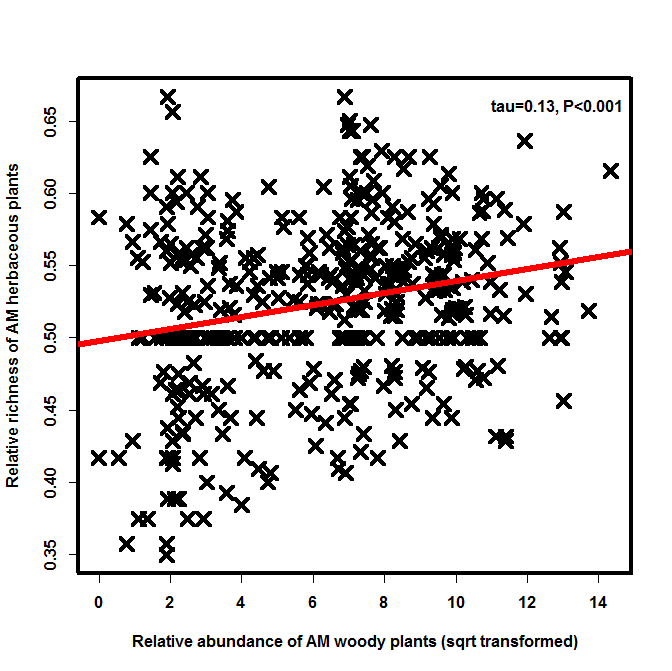


Fig. S4 Relationship between relative abundance of AM woody plants and relative richness of AM herbaceous plants in the understory of forests in the Weser- Elbe region in Germany. The red line was derived through median quartile regression. Statistics presented on the top right corner of the panel are based on a Kendall correlation test. Likely spatial dependencies were not considered in the test. For the specific figure the mycorrhizal status definitions from Hempel et al. (2013) were used (the two fifures are spectacularly similar – note how the relative position of the two plots with high relative AM herbaceous richnes but moderate (~2) woody plant abundance differ in the two figures).


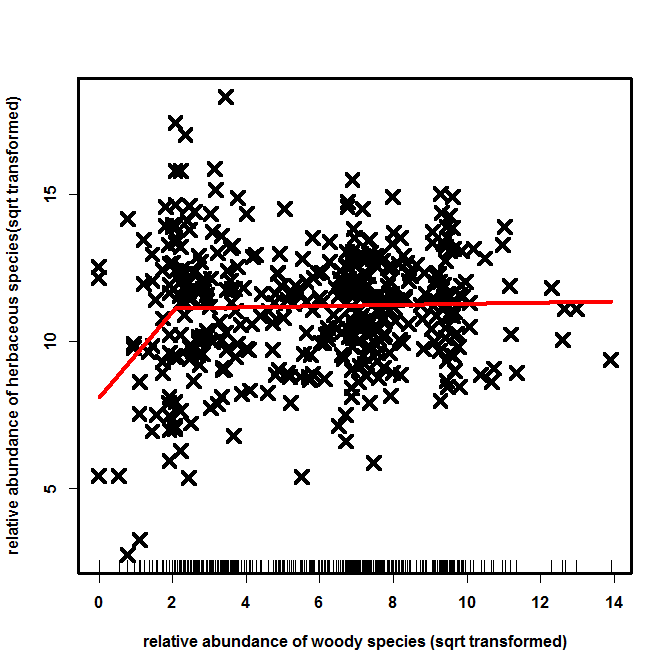

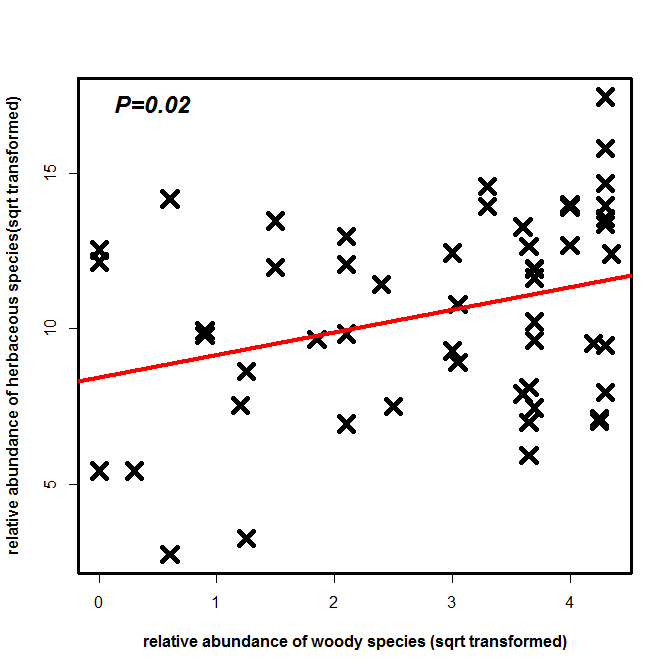


Fig S5. (a) segmented regression and (b) analysis of forest plots on the left side of the identified breakpoint between relative abundance of AM woody species and relative abundance of AM herbaceous plants. The analysis in panel (b) takes into consideration likely spatial dependencies and is based on a parametric linear model. For the specific figure the mycorrhizal status definitions from Hempel et al. (2013) were used. Note that there are only minor differences with regards to Fig. 3.

**Results Structural Equation Modeling**

**OUTPUT SEM: Model 1**

Model Chisquare = 0.2765313 Df = 1 Pr(>Chisq) = 0.5989838

AIC = 10.27653

BIC = -3.693761

Normalized Residuals

Min. 1st Qu. Median Mean 3rd Qu. Max.

0.0000 0.1200 0.1207 0.2247 0.3055 0.5252

R-square for Endogenous Variables

herbs3

0.4347

Parameter Estimates

Estimate Std Error z value Pr(>|z|)

ena 0.4107461 0.07111578 5.775737 7.661675e-09 herbs3 <--- pH3

duo 0.6150602 0.23897823 2.573708 1.006151e-02 herbs3 <--- woody3

a 1.9127304 0.37511729 5.099020 3.414174e-07 woody3 <--> woody3

b 5.6803328 1.11400491 5.099020 3.414174e-07 herbs3 <--> herbs3

c 21.5992420 4.23595986 5.099020 3.414174e-07 pH3 <--> pH3

**OUTPUT SEM: Model 2**

Model Chisquare = 0.558417 Df = 1 Pr(>Chisq) = 0.4548988

AIC = 10.55842

BIC = -3.411875

Normalized Residuals

Min. 1st Qu. Median Mean 3rd Qu. Max.

0.0000 0.1546 0.1634 0.3121 0.4182 0.7453

R-square for Endogenous Variables

herbs3

0.3994

Parameter Estimates

Estimate Std Error z value Pr(>|z|)

ena 4.798607e-02 8.908606e-03 5.386485 7.184911e-08 herbs3 <--- nutrients3

duo 5.789025e-01 2.455186e-01 2.357876 1.837982e-02 herbs3 <--- woody3

a 1.912730e+00 3.751173e-01 5.099020 3.414174e-07 woody3 <--> woody3

b 5.995508e+00 1.175816e+00 5.099020 3.414174e-07 herbs3 <--> herbs3

c 1.452791e+03 2.849157e+02 5.099020 3.414174e-07 nutrients3 <--> nutrients3

**OUTPUT SEM: Model 3**

Model Chisquare = 0.3865964 Df = 1 Pr(>Chisq) = 0.5340939

AIC = 10.3866

BIC = -3.224322

Normalized Residuals

Min. 1st Qu. Median Mean 3rd Qu. Max.

0.0000 0.1287 0.1360 0.2596 0.3480 0.6201

R-square for Endogenous Variables

herbs3

0.3994

Parameter Estimates

Estimate Std Error z value Pr(>|z|)

ena 4.798607e-02 0.01070681 4.481826 7.400706e-06 herbs3 <--- nutrients3

duo 5.789025e-01 0.29507667 1.961872 4.977744e-02 herbs3 <--- woody3

a 1.912730e+00 0.45083488 4.242641 2.209050e-05 woody3 <--> woody3

b 5.995508e+00 1.41315482 4.242641 2.209050e-05 herbs3 <--> herbs3

c 1.452791e+03 342.42610878 4.242641 2.209050e-05 nutrients3 <--> nutrients3

**OUTPUT SEM: Model 4**

Model Chisquare = 1.378634 Df = 1 Pr(>Chisq) = 0.240334

AIC = 11.37863

BIC = -2.591658

Normalized Residuals

Min. 1st Qu. Median Mean 3rd Qu. Max.

0.0000 0.2073 0.2280 0.4747 0.6380 1.1660

R-square for Endogenous Variables

herbs3

0.3645

Parameter Estimates

Estimate Std Error z value Pr(>|z|)

ena 4.2837258 0.84480659 5.070659 3.964413e-07 herbs3 <--- dens.ratio3

duo 0.5094747 0.25129153 2.027425 4.261898e-02 herbs3 <--- woody3

a 1.9127304 0.37511729 5.099020 3.414174e-07 woody3 <--> woody3

b 6.2807690 1.23176014 5.099020 3.414174e-07 herbs3 <--> herbs3

c 0.1692368 0.03319007 5.099020 3.414174e-07 dens.ratio3 <--> dens.ratio3

**OUTPUT SEM: Model 5**

Model Chisquare = 5.920947 Df = 2 Pr(>Chisq) = 0.0517944

AIC = 21.92095

BIC = -2.019637

Normalized Residuals

Min. 1st Qu. Median Mean 3rd Qu. Max.

0.0000 0.0000 0.7453 0.7376 1.0220 2.3360

R-square for Endogenous Variables

dens.ratio3 herbs3

0.0262 0.4962

Parameter Estimates

Estimate Std Error z value Pr(>|z|)

ena 3.193949e+00 7.229322e-01 4.418048 9.959630e-06 herbs3 <--- dens.ratio3

duo 4.557233e-01 2.150395e-01 2.119254 3.406899e-02 herbs3 <--- woody3

tria 4.811397e-02 4.070632e-02 1.181978 2.372145e-01 dens.ratio3 <--- woody3

tessera 3.727997e-02 7.699924e-03 4.841602 1.287964e-06 herbs3 <--- nutrients3

a 1.912730e+00 3.751173e-01 5.099020 3.414174e-07 woody3 <--> woody3

b 4.478982e+00 8.784006e-01 5.099020 3.414174e-07 herbs3 <--> herbs3

c 1.648090e-01 3.232169e-02 5.099020 3.414174e-07 dens.ratio3 <--> dens.ratio3

d 1.452791e+03 2.849157e+02 5.099020 3.414174e-07 nutrients3 <--> nutrients3
